# Giant eccrine Poroma of the posterior scalp treated with Mohs surgery

**DOI:** 10.1016/j.jdcr.2026.09.007

**Published:** 2026-09-10

**Authors:** Brian Goldspiel, Shivam Zaver, Mia Do, Christopher Richter, Leela Raj, Rosalie Elenitsas, Junqian Zhang, Leo Wang

**Affiliations:** a Medical Scientist Training Program, Perelman School of Medicine, University of Pennsylvania, Philadelphia, Pennsylvania; b Department of Dermatology, Perelman School of Medicine, University of Pennsylvania, Philadelphia, Pennsylvania

**Keywords:** head and neck tumor, mohs micrographic surgery (MMS), Poroma

## Introduction

Eccrine poromas are uncommon, benign adnexal tumors originating from the intra-epidermal eccrine sweat gland ducts and typically arise on hairless acral surfaces.^1^ Compromising only a small proportion of all cutaneous lesions, they are frequently underreported and the literature lacks a complete description of the heterogeneity of these tumors and their presentations. Here, we present a poroma in a young and otherwise healthy patient remarkable for its location, size, and depth of invasion. We demonstrate Mohs micrographic surgery (MMS) as a highly effective treatment for managing clinically atypical poromas, particularly large lesions and those occurring on sensitive body surfaces such as the scalp and neck. These findings highlight the importance of considering eccrine tumors in the differential diagnosis of large scalp tumors and demonstrate the need for histopathology in establishing the diagnosis of these uncommon lesions.

## Case presentation

A 39-year-old male with no relevant past medical history presented to our outpatient dermatology clinic with a 3-year history of an enlarging, painful mass on his occipital scalp and neck. The patient was initially evaluated in the emergency department for a presumed abscess; however, the lesion persisted and continued to enlarge following incision and drainage, prompting dermatology consultation. Skin examination revealed a 5 centimeter rubbery, subcutaneous mass with an overlying central ulcerated black nodule (Fig 1, *A*). Ultrasound was performed, which showed a complex, mixed cystic and solid lesion abutting the calvaria, with prominent blood flow and multiple echogenic foci (Fig 1, *B*). These findings were confirmed on CT imaging, which demonstrated the tumor abutting the trapezius muscle near the occiput (Fig 1, *C* and *D*). Notably, the patient had no prior history of malignancy nor immunosuppression, and no familial history of skin cancer.

**Fig 1:**
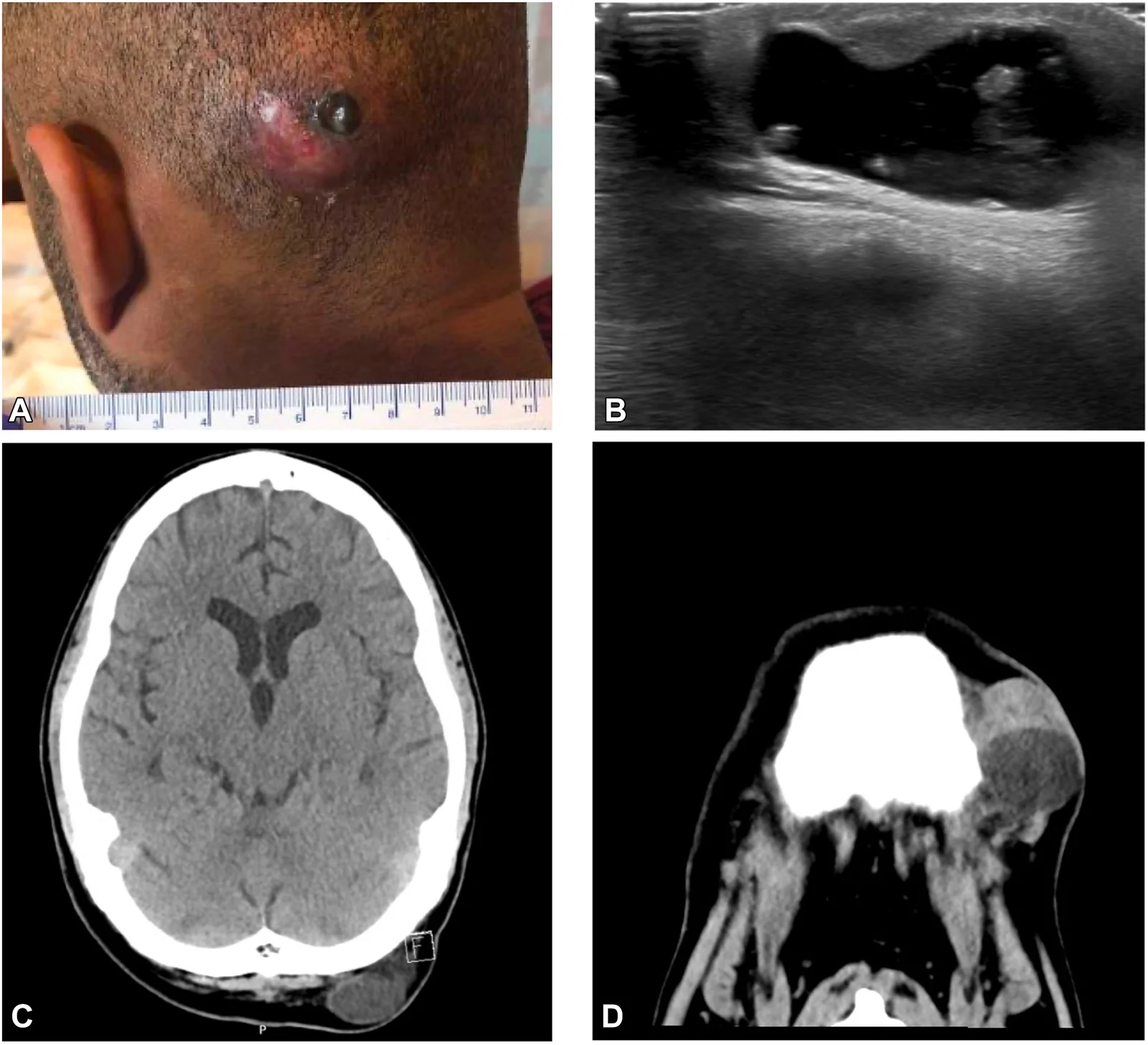
**A,** Posterior scalp mass prior to diagnostic biopsy. Exam demonstrates a 5 cm rubbery, subcutaneous mass with an overlying small central ulcerated black nodule. **B-D,** Ultrasound and CT imaging performed in this region demonstrates a complex, mixed cystic and solid lesion with multiple echogenic foci abutting the occipital bone and trapezius muscle at the posterior neck.

An incisional biopsy demonstrated a lobular proliferation of basaloid, bland-appearing cells at the base of the epidermis descending into the dermis (Fig 2, *A*). Focally, duct-like structures were identified and brown pigment was noted within the lesion (Fig 2, *B*). While porocarcinoma was considered given the tumor’s unusual size and deep extension, the lesion lacked cytologic atypia, frequent mitotic figures, necrosis, an infiltrative growth, or destruction of normal structures, and was therefore most consistent with a pigmented eccrine poroma.

**Fig 2:**
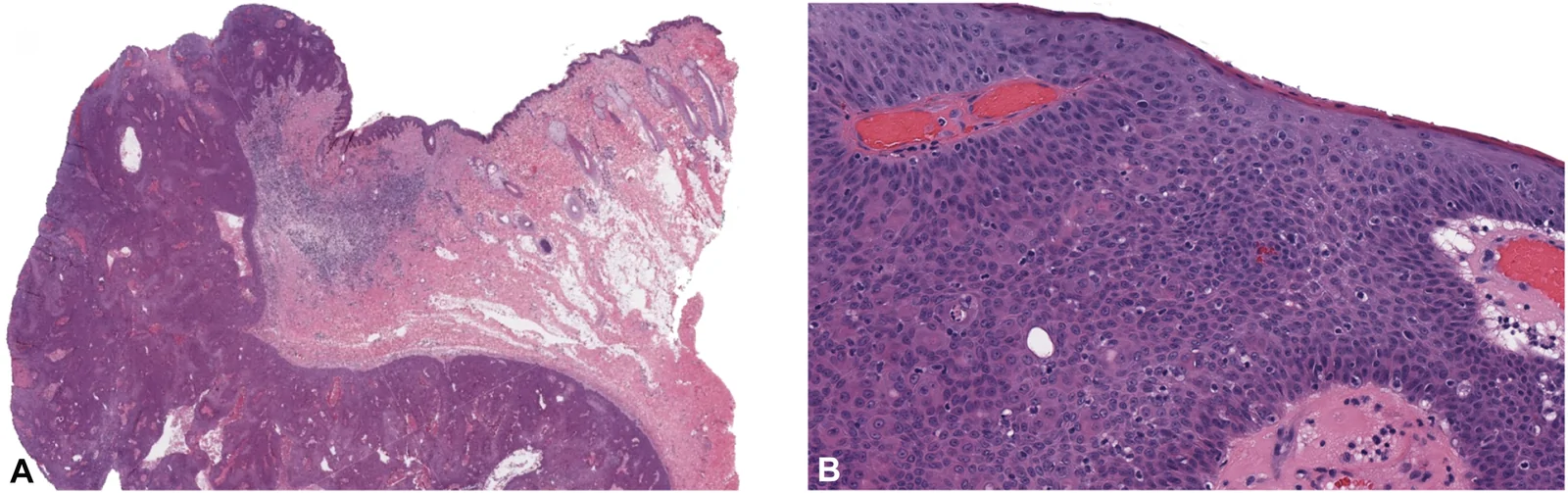
**A,** Biopsy of the mass shows a lobular proliferation of basaloid cells from the base of the epidermis descending into the dermis, H&E, 1×. **B,** Focal duct-like structures and brown pigment are present, H&E, 20×.

Due to the lesion’s considerable size and anatomic location, definitive surgical management with Mohs micrographic surgery (MMS) was recommended. Intraoperatively, the tumor was found to extend deep into the investing fascia overlying the superior fibers of the trapezius near its cranial origin from the occipital bone and the posterior neck (Fig 3). The lesion was cleared after 1 stage of MMS and repaired with a synthetic skin substitute without subsequent delayed skin grafting.

**Fig 3:**
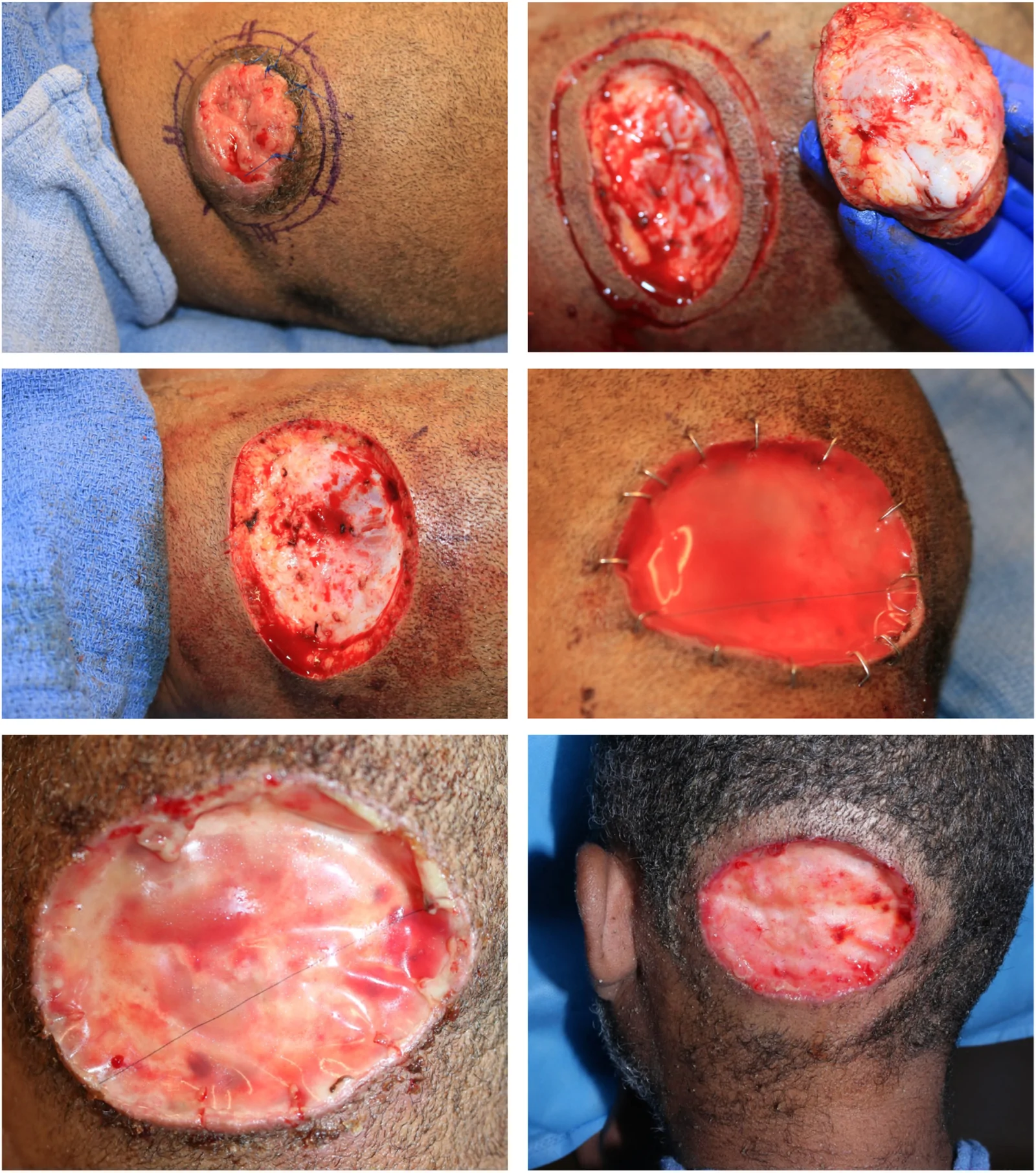
Mohs micrographic surgery was employed to remove the tumor. The Mohs layer extended to the investing fascia of the trapezius muscle. The tumor was successfully removed with clear microscopic margins after 1 stage and the resulting defect was allowed to heal by secondary intention with the aid of Integra bilayer wound matrix. At follow up, the patient reported only mild residual pain with continued healing.

## Discussion

Poromas are rare cutaneous neoplasms that arise from eccrine sweat glands.^1^ These tumors remain a diagnostic challenge in part because of their rarity, comprising less than 1% of all primary cutaneous lesions, as well as their heterogeneity in presentation.^1^ While these tumors are typically associated with palmoplantar skin, they can also be found on other body sites, including an estimated 20% on the head and neck.^2^^,^^3^ Given their relatively unusual location, head and neck poromas are frequently underdiagnosed compared to those found on acral surfaces and, because of this, may be more common than the literature reports.^4^ While uncommon, local invasion of tissue and malignant transformation may occur with these tumors, making prompt diagnosis critical for preventing these outcomes.^5^ In our case, intraoperative MMS findings demonstrated tumor invasion into the investing fascia of the trapezius muscle.

Risk factors for developing poromas are not completely understood, but recent evidence suggests a role for the immune system. Immunosuppression in the setting of chemotherapy or malignancies has been associated with an increased risk of developing poromas, although this association is not well established.^6^ Overall, most poromas are identified in patients with seemingly no predisposing risk factors.^1^ When they do occur in healthy individuals, the average age of these patients is in the sixth to eighth decade of life.^1^ This case illustrates that even healthy, young patients without predisposing conditions can develop unusual presentations of these tumors, underlying the need for including poromas on the differential diagnosis for head and neck masses.

Head and neck poromas are typically small, averaging approximately 1 cm in diameter, and the largest benign poroma reported to date measured 6 cm on the foot.^4^^,^^7^ In comparison, the present lesion is the largest head and neck poroma reported and is among the largest benign poromas documented in the literature. Given the lesion’s unusually large size, clinically ill-defined subcutaneous extension, complex anatomic location on the scalp and neck, and proximity to critical structures, we opted for Mohs micrographic surgery over a standard surgical excision. This allowed for complete peripheral and deep margin assessment while maximizing tissue preservation for a cosmetically and functionally sensitive area.

While surgical removal of poromas has been shown to be an effective method of treatment for these tumors, its utility for all cases has not been uniformly accepted.^8^ Removal of poromas prevents the potentially rare complication of malignant transformation into porocarcinoma – however, this risk remains controversial with a 2001 study suggesting that less than 20% of porocarcinomas arise from benign poromas.^5^ As in this case, surgical excision of even benign poromas in unusual locations, such as those on the head and neck, may be particularly important given the theoretical risk of invasion of local structures by the primary lesion. Previous case reports for poromas have supported the idea that MMS may be used to successfully excise these lesions, including those found on the head and neck, and have led to good long-term outcomes.^9^ However, these case reports have not included patients such as this one with lesions on the posterior scalp and neck nor lesions of this size. Therefore, this report demonstrates Mohs to be a broadly useful method for treating unusually sized and located lesions and supports the role for complete surgical excision of poromas.

## Conflicts of interest

None disclosed.
